# Vietnam, a Hotspot for Chromosomal Diversity and Cryptic Species in Black Flies (Diptera: Simuliidae)

**DOI:** 10.1371/journal.pone.0163881

**Published:** 2016-10-03

**Authors:** Peter H. Adler, Hiroyuki Takaoka, Mohd Sofian-Azirun, Van Lun Low, Zubaidah Ya’cob, Chee Dhang Chen, Koon Weng Lau, Xuan Da Pham

**Affiliations:** 1 Department of Agricultural and Environmental Sciences, Clemson University, Clemson, South Carolina 29634, United States of America; 2 Institute of Biological Sciences, University of Malaya, Kuala Lumpur 50603, Malaysia; 3 Tropical Infectious Diseases Research & Education Centre, University of Malaya, Kuala Lumpur 50603, Malaysia; 4 National Agency in Southern Region, Ministry of Science and Technology, Ho Chi Minh City, Vietnam 700000; Universita degli Studi di Roma La Sapienza, ITALY

## Abstract

The increasing attention on Vietnam as a biodiversity hotspot prompted an investigation of the potential for cryptic diversity in black flies, a group well known elsewhere for its high frequency of isomorphic species. We analyzed the banding structure of the larval polytene chromosomes in the *Simulium tuberosum* species group to probe for diversity beyond the morphological level. Among 272 larvae, 88 different chromosomal rearrangements, primarily paracentric inversions, were discovered in addition to 25 already known in the basic sequences of the group in Asia. Chromosomal diversity in Vietnam far exceeds that known for the group in Thailand, with only about 5% of the rearrangements shared between the two countries. Fifteen cytoforms and nine morphoforms were revealed among six nominal species in Vietnam. Chromosomal evidence, combined with available molecular and morphological evidence, conservatively suggests that at least five of the cytoforms are valid species, two of which require formal names. The total chromosomal rearrangements and species (15) now known from the group in Vietnam far exceed those of any other area of comparable size in the world, supporting the country’s status as a biodiversity hotspot. Phylogenetic inference based on uniquely shared, derived chromosomal rearrangements supports the clustering of cytoforms into two primary lineages, the *Simulium tani* complex and the Southeast Asian *Simulium tuberosum* subgroup. Some of these taxa could be threatened by habitat destruction, given their restricted geographical distributions and the expanding human population of Vietnam.

## Introduction

Vietnam moved to center stage in the 1990s as a hotspot for biodiversity and endemism when new species of large mammals were discovered [1]. These discoveries were not isolated examples of Vietnam’s remarkable biodiversity. New species have been discovered in nearly every investigated group of animals and plants in Vietnam, highlighting the country’s standing as 25^th^ in the world in species richness [2] despite ranking 65^th^ in total area. The wealth of Vietnam’s biodiversity derives from a complex climatic and geological history, significant elevational (0–3143 m asl) and latitudinal gradients (8.4–23.4° N), and a subtropical-tropical setting with diverse ecoregions [2–4].

Invertebrate diversity in Vietnam is rich but woefully underexplored. Two of the best-known groups, butterflies and mosquitoes, provide a general index of richness in the country. More than 1100 species of butterflies [5] and about 226 species of mosquitoes [6] are known from Vietnam—more than 6% of each group’s total world fauna. The insect diversity in Vietnam has been explored largely through conventional morphological approaches. The actual extent of biodiversity is probably far greater when cryptic species are considered [7]. Taking a lead from amphibian studies, which have recognized two to six times the number of each putative species of frog in Vietnam [8,9], biologists might expect comparable cryptic species richness in insects.

Among the insect groups best known for repetitive discoveries of cryptic species are the black flies (Simuliidae) and mosquitoes [10,11]. A cryptic species of mosquito, for example, was discovered in Vietnam when malarial vectors were investigated [12], and three additional species of black flies were revealed among two nominal species when molecular techniques were applied [13]. The intricate banding patterns of polytene (giant) chromosomes provide a time-tested means of revealing cryptic species of black flies through evidence of reproductive isolation [14]. The taxonomic framework for the family Simuliidae now rests in significant part on characters of the polytene chromosomes [11,14].

Indications that Vietnam is a hotspot for biodiversity in the Simuliidae are based on recent surveys in three of the 58 provinces, which increased the country’s number of known species to 46, including 22 (48%) described as new [15–18]. Our objective was to explore the biodiversity that might further be revealed in the macrogenome of a single species group of black flies in Vietnam. We selected the *Simulium tuberosum* species group, based on an opportunity to compare our findings with those of a molecular study of two nominal species in the group in Vietnam [13] and with the extensive cryptic taxa discovered in the group in Thailand [19]. The *Simulium tuberosum* group is a well-defined clade [20] of more than 50 nominal species distributed across the Holarctic Region deep into the Oriental Region [21]. The Holarctic namesake (*Simulium tuberosum sensu stricto*) for the group provided one of the earliest examples of chromosomal discovery of cryptic species in the family Simuliidae [22].

## Materials and Methods

### Ethics statement

All samples were collected on public land with access from public roads. No permissions were required to access sites or collect material, and the collections did not involve endangered or protected species.

### Collection and preparation of material

Larvae and pupae were collected with forceps from substrates in 16 streams in Vietnam, spanning more than 1150 km of the country’s length, plus 2 streams in Malaysia to aid species identifications (Table 1). They were fixed in ethanol or (larvae only) in 1:3 glacial acetic acid:95% ethanol (Carnoy’s fixative). Adults were allowed to emerge from additional pupae to facilitate morphological identifications. Larvae in Carnoy’s fixative were sorted into morphotaxa, based on their key characters and those of associated life stages [18]. Polytene chromosomes were prepared according to standard Feulgen-staining procedures [23].

**Table 1:** Collection information for larvae of the *Simulium tuberosum* group in Vietnam.

Site No.	Location	Latitude Longitude	Elevation(m asl)	Date	Morphoform (larvae)^1^	Cytoform (females:males)^2^
1	Lam Dong Province, Suoi Vang Natural Forest, Dalat-1	11°59'26"N 108°22'06"E	1443	22 April 2014	*S*. *tani* ‘b’ (22)*	*S*. *tani* ‘M’ (8:14)
2	Lam Dong Province, Dalat 4	12°05'49"N 108°22'36"E	1746	23 April 2014	*S*. *congi* (1)	*S*. *congi* (1:0)
3	Lam Dong Province, Dinh Kno, Lac Duong, Dalat-5	12°06'07''N 108°22'03''E	1722	24 April 2014	*S*. *congi* (4)	*S*. *congi* (2:1), *S*. *doipuiense* ‘C’ (1:0)
4	Lam Dong Province, Dalat -9	12°10'56"N 108°40'48"E	1452	24 April 2014	*S*. *xuandei* (9)	*S*. *xuandei* (6:3)
5	Thua Thien Hue Province, Luoi-1	16°18'16''N 107°12'48''E	629	24 Feb 2014	*S*. *tani* ‘a’ (60)	*S*. *tani* ‘B2’ (17:20 + 11^3^), *S*. *tani* ‘N’ (5:6 + 1^3^)
6	Thua Thien Hue Province, Bach Ma-2	16°11'43''N 107°51'28''E	1274	23 Feb 2014	*S*. *cavum* (14), *S*. *rufibasis* (10)	*S*. *yuphae* ‘A’ (4:10), *S*. *doipuiense* ‘D’ (4:6)
7	Thua Thien Hue Province, Bach Ma-3	16°11'45''N 107°50'56''E	1187	23 Feb 2014	*S*. *cavum* (3), *S*. *rufibasis* (4)	*S*. *yuphae* ‘A’ (2:1), *S*. *doipuiense* ‘D’ (2:2)
8	Thua Thien Hue Province, Bach Ma-7	16°13'56''N 107°51'19''E	434	23 Feb 2014	*S*. *cavum* (2)*	*S*. *yuphae* ‘A’ (0:2)
9	Vinh Phuc Province, Tam-Dao (st.-1)	21°27'30"N 105°38'16"E	975	8 Nov 2013	*S*. *brevipar* (4)	*S*. *brevipar* ‘B’ (0:2), *S*. *yuphae* ‘A’ (2:0)
10	Lao Cai Province, Sapa-1	22°22'05''N 103°47'34''E	1680	20 Dec 2014	*S*. ‘Sapa’ (2)	*S*. *yuphae* B’ (0:2)
11	Lao Cai Province, Sapa-2	22°21'43''N 103°47'19''E	1750	20 Dec 2014	*S*. *rufibasis* ‘B’ (8)	*S*. *doipuiense* ‘A’ (3:4), *S*. *rufibasis* ‘B’ (1:0)
12	Lao Cai Province, Sapa-8	22°22'23''N 103°45'25''E	1728	20 Dec 2014	*S*. *rufibasis* ‘B’ (14)	*S*. *brevipar* ‘C’ (1:0), *S*. *doipuiense* ‘A’ (1:0), *S*. *rufibasis* ‘B’ (2:9 + 1^4^)
13	Lao Cai Province, Sapa-13	22°18'48''N 103°53'10''E	1105	21 Dec 2014	*S*. *rufibasis* ‘B’ (28)*	*S*. *doipuiense* ‘A’ (8:17 + 2^5^), *S*. *rufibasis* ‘B’ (0:1)
14	Lao Cai Province, Sapa-15	22°18'24''N 103°53'43''E	999	21 Dec 2014	*S*. *rufibasis* ‘B’ (10)*	*S*. *doipuiense* ‘A’ (3:7)
15	Lao Cai Province, Sapa-21	22°23'03''N 103°50'59''E	1315	22 Dec 2014	*S*. *rufibasis* ‘B’ (61)	*S*. *doipuiense* ‘A’ (25:27), *S*. *doipuiense* ‘E’ (6:2), *S*. *doipuiense* ‘F’ (1:0)
16	Lao Cai Province, Sapa-26	22°24'50''N 103°53'55''E	708	23 Dec 2014	*S*. *rufibasis* ‘B’ (16)*	*S*. *doipuiense* ‘A’ (7:8 + 1^6^)
17	Malaysia, Cameron Highland, Brinchang (2)	04°31'28"N 101°23'20"E	1813	28 Jan 2011	*S*. *brevipar* (7)	*S*. *brevipar* ‘A’ (3:4)
18	Malaysia, Tapah, CHS5	04°22'13"N 101°21'31"E	711	29 Jan 2011	*S*. *brevipar* (1)	*S*. *brevipar* ‘A’ (0:1)

^1^ Number of morphologically identified larvae whose band sequences were analyzed entirely. An asterisk (*) indicates that chromosomally prepared larvae from the following sites, whose banding patterns could not be evaluated completely, were not included in any sample sizes or analyses: Site 1 (3 larvae), Site 8 (5 larvae), Site 13 (6 larvae), Site 14 (3 larvae), and Site 16 (1 larva).

^2^ Number of female and male larvae fully analyzed chromosomally.

^3^ + gender undetermined; these 11 larvae of ‘B2’ and 1 larva of ‘N’ were infected with mermithid nematodes.

^4^ + gender undetermined; 1 larva was infected with a microsporidian parasite.

^5^ + gender undetermined; 2 larvae were infected with non-mermithid parasites of the clade Nematoida.

^6^ + gender undetermined; 1 larva was infected with a chytrid fungus resembling *Coelomycidium simulii*.

Carcasses of all chromosomally examined larvae and photographic negatives of chromosomes are deposited in the Clemson University Arthropod Collection. Additional larvae and associated life stages are deposited in the Institute of Biological Sciences, Faculty of Science, University of Malaya, Kuala Lumpur, Malaysia.

### Chromosomal mapping and analyses

Chromosomal mapping procedures, conventions, and terminology follow established procedures [19, 24]. Chromosomal banding patterns of larvae were compared with the standard banding sequence of the subgenus *Simulium* and the *Simulium tuberosum* group [19,24]. Section numbers on our maps follow those for the *S*. *tuberosum* group [19]. Inversions discovered in our material, which are shared with species previously known chromosomally in Southeast Asia [19], are given the same number. Newly discovered inversions are numbered to follow the last-used number in each chromosome arm in previous treatments [24] of the *S*. *tuberosum* species group. Fixed inversions within a cytoform are italicized; polymorphic inversions are not. Each heteroband (hb; thickened band relative to the standard), heterochromatic block (hc; insertion of heterochromatin between existing bands), and finer band insertion (in) is coded by the arm and section number in which it occurs (e.g., IS 13hb, IIIL 100hc, and IIIL 85in, respectively). All chromosomal rearrangements were indicated with precise locations and breakpoints on our maps.

We use the following previously applied [25] definition of cytoform: a chromosomally distinct entity recognizable at an individual or a population level, without regard to whether the entity is part of a larger breeding population (cytotype) or is reproductively isolated (cytospecies). New cytoforms of the *S*. *doipuiense* and *S*. *tani* complexes were named to follow the last-recognized cytoforms (‘B’ and ‘L’, respectively) [21]. New cytoforms of the nominal species *S*. *brevipar*, *S*. *rufibasis*, and *S*. *yuphae*, not previously known to contain cytoforms, were each designated Cytoform ‘B’ (and Cytoform ‘C’ for *S*. *brevipar*), while the original chromosomally studied population of each was assigned, retrospectively, to Cytoform ‘A’.

We inferred a phylogeny based on uniquely shared, derived chromosomal rearrangements, primarily inversions, from the polytene complement. We used a two-step procedure [24]. Briefly, we first resolved all rearrangements in our material, relative to the *Simulium* subgeneric standard for the IS, IL, IIL, and IIIS arms [26] and the IIS and IIIL arms [19,27]. To provide directionality, we then rooted the phylogeny by resolving the subgeneric standard where possible, particularly for the entire IIIL arm, relative to the common sequences [28,29] in two outgroups, *Simulium* (*Boophthora*) *erythrocephalum* and *Simulium* (*Psilozia*) *vittatum*.

## Results

The banding patterns of 280 larvae (including 8 from Malaysia) were analyzed completely; the chromosomes of 18 additional larvae (6.0%) were not of sufficient quality for full resolution, and were not included in any tabulations or analyses. A total of 88 chromosomal rearrangements, primarily (86.4%) paracentric inversions, but also differential band expressions (13.6%), were discovered in Vietnamese material (plus 1 additional novel inversion in our Malaysian samples), relative to the standard sequence for the *S*. *tani* complex and the Southeast Asian *S*. *tuberosum* species subgroup. Rearrangements were concentrated (69.3%) in the IIIL arm. Chromocenters, ectopic pairing of centromeres, and supernumerary (B) chromosomes were absent.

The cytoforms fell into 2 previously defined [19] lineages: the *Simulium tani* complex and the Southeast Asian *Simulium tuberosum* subgroup. We describe each cytoform under its chromosomally assigned name. Table 1 links the initial morphological identification of each cytoform with its chromosomal designation, and Table 2 summarizes the diagnostic information for each cytoform in Vietnam (plus one in Malaysia).

**Table 2:** Summary of diagnostic chromosomal rearrangements for cytoforms of the *Simulium tuberosum* group in Vietnam.

Cytoforms	Larvae (*n*)	Fixed inversions^1^	Common^2^ autosomal polymorphisms	Sex-linked rearrangements	Notes
***tani* complex**					
*tani* ‘B2’	48	none	none	none	IL-2 is absent
*tani* ‘M’	22	*IL-2*	IIIL-47	none	
*tani* ‘N’	12	*IIIL-5*, *IIIL-54*, *IIIL-55*	IL-14	none	
*xuandei*	9	*IS-23*, *IL-2*, *IIIL-34*, *IIIL-49*	IIIL-50, IIIL-51, IIIL-52	none	
***tuberosum* subcomplex**					
*brevipar* ‘A’	8	*IIIL-13*	IS-26	none	Malaysian sample
*brevipar* ‘B’	2	*IIIL-13*, *IIIL-81*, *IIIL-82*	none	IIIL-83?	possibly Y linked
*brevipar* ‘C’	1	*IIIL-13*, *IIIL-84*, *IIIL-85*, *IIIL-86*	none	?	
*congi*	4	*IIIL-13*, *IIIL-71*, *IIIL-72*, *IIIL-80*	none	none	
*doipuiense* ‘A’	113	*IIIL-11*, *IIIL-13*	none	none or rare	autosomal inversions disproportionately in linkage groups
*doipuiense* ‘C’	1	*IS-32*, *IL-16*, *IIIL-11*, *IIIL-13*, *IIIL-79*	none	?	
*doipuiense* ‘D’	14	*IIIL-11*, *IIIL-13*, *IIIL-79*	none	none	
*doipuiense* ‘E’	8	*IIIL-11*, *IIIL-13*	none	IIIL-68, IIIL-69, IIIL-70, 100hb1	probably X linked
*doipuiense* ‘F’	1	*IIIL-11*, *IIIL-13*, *IIIL-87*, *IIIL-88*, *IIIL-89*	none	?	
*rufibasis* ‘B’	14	*IIIL-8*, *IIIL-11*, *IIIL-13*	IIIL-12, IIIL-60	IS-27, IS-28, IS-29, IS-31, IS 13hb	differentiated X and Y chromosomes
*yuphae* ‘A’	21	*IIIL-12*, *IIIL-13*	none	none	
*yuphae* ‘B’	2	*IIIL-12*, *IIIL-13*	none	IIIL-57?, IIIL-58?	possibly sex linked

^1^ All members of the *S*. *tani* complex had 3 inversions in IL, 6 inversions in IIS, 3 inversions in IIL, and 3 inversions in IIIL (*IIIL-1*, *IIIL-2*, and *IIIL-3*); all members of the Southeast Asian *S*. *tuberosum* subgroup had *IL-1*, *IL-tuberosum*, 4 inversions in IIS, 3 inversions in IIL, and *IIIL-1* [19].

^2^ Common = frequency of inverted homologues > 0.33.

### *Simulium tani* species complex

**Fig 1:**
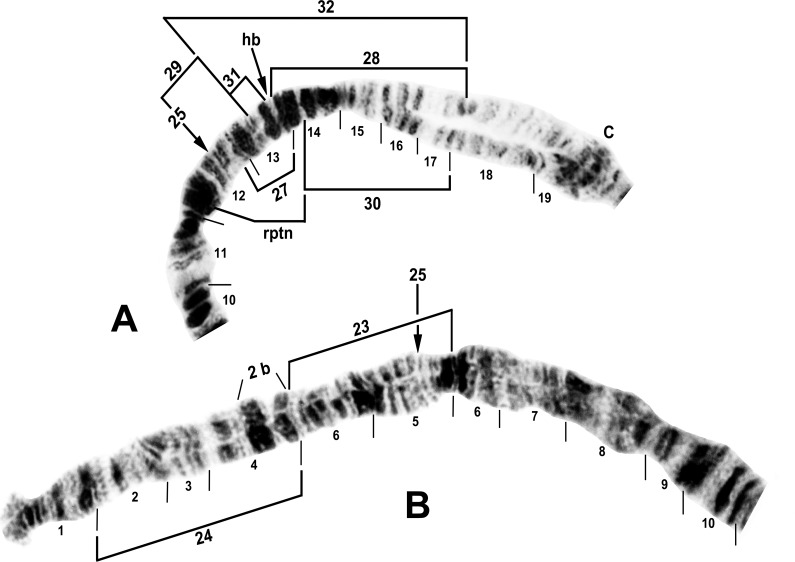
IS arm (male larvae) of *Simulium tani* group from Vietnam. A. *Simulium tani* ‘B2’ (Site 5), showing *Simulium* subgeneric standard sequence, with limits of polymorphic inversions IS-25 and IS-27–IS-32 of various cytoforms indicated by brackets. C = centromere, hb = location of heteroband, rptn = location of repatterned sections. B. *Simulium xuandei* (Site 4), showing the *IS-23* sequence. Limits are indicated for breakpoints of polymorphic inversions IS-24 (bracket) of *S*. *xuandei* and IS-25 (arrows) of *S*. *tani* ‘B2’; 2 b = 2 blocks marker.

**Fig 2:**
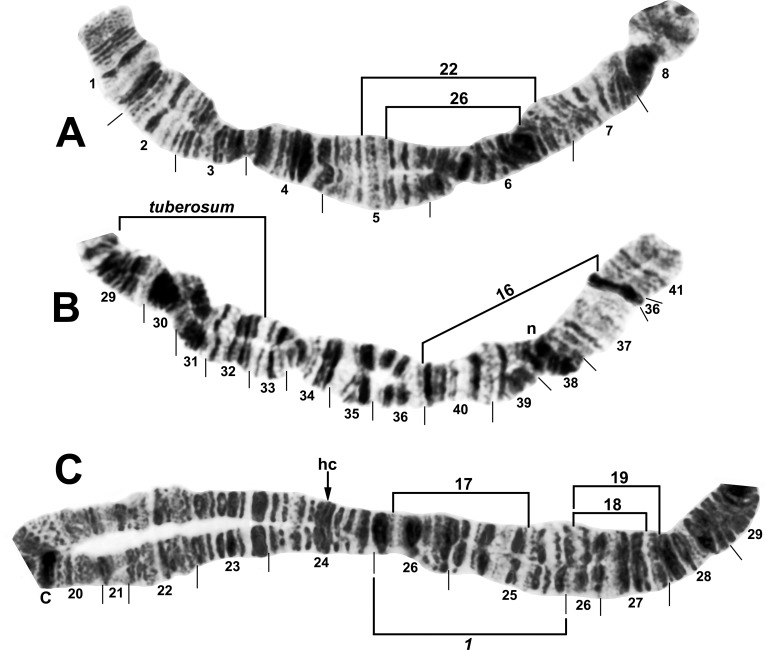
Chromosome I (female larva) of *Simulium tuberosum* group from Vietnam. A. Distal half of IS (Site 12) of *Simulium brevipar* ‘C’, with limits of autosomal polymorphisms IS-22 of *S*. *tani* ‘M’ and IS-26 of *S*. *brevipar* ‘A’ indicated by brackets. B. Distal portion of IL (Site 3) of *S*. *doipuiense* ‘C’, showing the *IL-tuberosum* sequence characteristic of all members of the Southeast Asian *S*. *tuberosum* subgroup, plus the *IL-16* sequence; n = neck marker. **C.** Basal portion of IL (Site 15) of *S*. *doipuiense* ‘A’, showing the *IL-1* sequence common to all members of the Southeast Asian *S*. *tuberosum* subgroup except *S*. *weji*; the limits of polymorphic inversions IL-17, IL-18, and IL-19 of *S*. *doipuiense* cytoforms are indicated by brackets. C = centromere, hc = location of heterochromatic block in *S*. *doipuiense* ‘D’.

**Fig 3:**
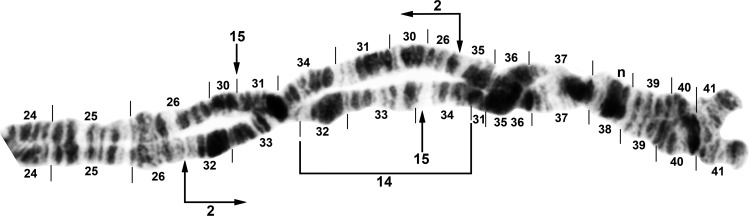
Distal half of IL arm (male larva) of *Simulium tani* ‘N’ from Vietnam (Site 5). IL-2 and IL-14 are shown heterozygously (on opposite homologues). Breakpoints of IL-15 of *S*. *tani* ‘B2’ are indicated by arrows; n = neck marker.

**Fig 4:**
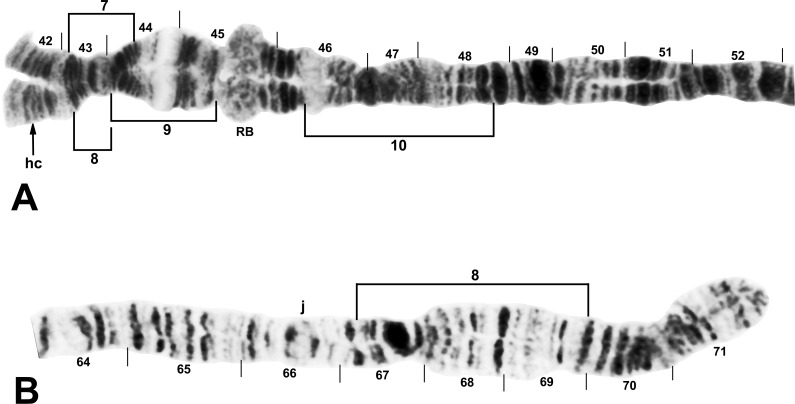
Chromosome II (female larvae) of *Simulium tuberosum* group from Vietnam. A. IIS arm (except extreme base) of *Simulium doipuiense* ‘A’ (Site 15), with limits of its autosomal polymorphisms (IIS-7–IIS-10) indicated by brackets; hc = insertion point for heterochromatic block, RB = ring of Balbiani. B. Distal portion of IIL arm of *Simulium tani* ‘N’ (Site 5), with breakpoints of polymorphic inversion IIL-8 indicated by brackets; j = jagged marker.

**Fig 5:**
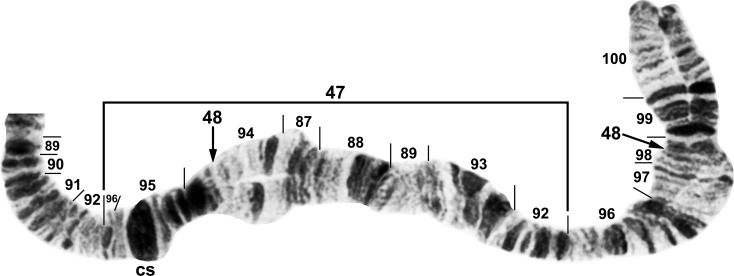
Distal portion of IIIL arm (male larva) of *Simulium tani* ‘M’ from Vietnam (Site 1). Polymorphic inversion IIIL-47 is present (homozygous); breakpoints of IIIL-48, which occurs independently of IIIL-47, are indicated by arrows; cs = cup and saucer marker.

**Fig 6:**
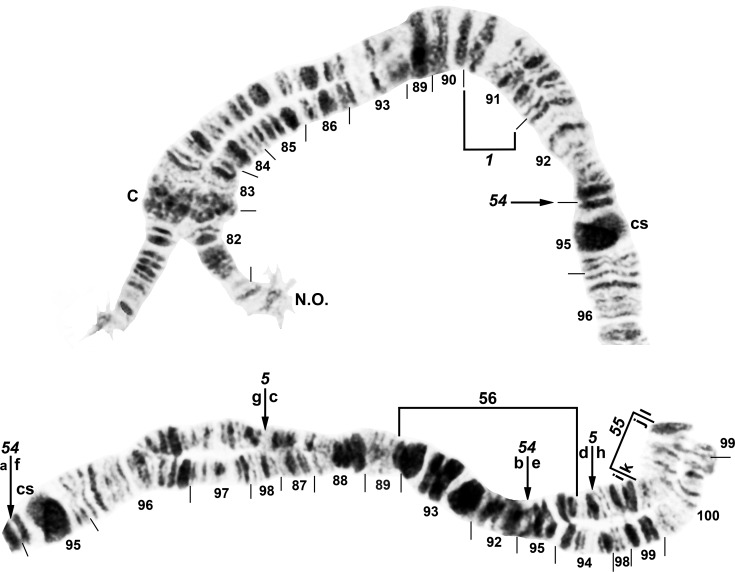
IIIL arm (male larva) of *Simulium tani* ‘N’ from Vietnam (Site 5). Fixed inversions *IIIL-1*, *IIIL-5*, *IIIL-54*, and *IIIL-55* are present. Breakpoints of polymorphic inversion IIIL-56 are indicated by a bracket. The sequence of the *S*. *tani* standard [19] can be obtained by alphabetically ordering the fragments indicated by the letters a–l. C = centromere, cs = cup and saucer marker, N.O. = nucleolar organizer.

**Fig 7:**
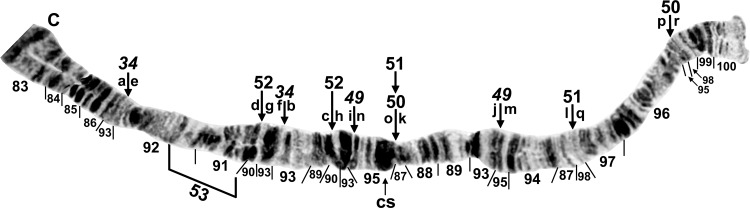
IIIL arm (male larva) of *Simulium xuandei* from Vietnam (Site 4). The most common sequence (65% of homologues) is shown: *IIIL-34*,*49*,50,51,52; arrows show breakpoints of these 5 inversions. Breakpoints of polymorphic inversion IIIL-53 (not present) are indicated by a bracket. The sequence of the *S*. *tani* standard [19] can be obtained by alphabetically ordering the fragments indicated by the letters a–r. C = centromere, cs = cup and saucer marker.

Four cytoforms were found among 3 morphoforms in the *S*. *tani* complex (Table 1). Relative to the *Simulium* subgeneric standard map, all 4 cytoforms shared the typical fixed sequence for the *S*. *tani* complex of Southeast Asia, which included 3 inversions in IL, 6 inversions in IIS, 3 inversions in IIL, and 3 inversions in IIIL (*IIIL-1*, *IIIL-2*, and *IIIL-3*) [19]. These inversions, therefore, are not repeated in descriptions of the taxa presented here, nor in Table 3. Figs 1–7 show all rearrangements discovered in our material of the *S*. *tani* complex.

**Table 3:** Frequency of chromosomal homologues with rearrangements in the *Simulium tani* complex in Vietnam.

CR^1^	Cytoform (*n*)			
	*tani* B2 (48)	*tani* M (22)	*tani* N (12)	*xuandei* (9)
IS-22		0.02		
*IS-23*				1.00
IS-24				0.06
IS-25	0.01			
IL-2		1.00	0.08	1.00
IL-14			0.92	
IL-15	0.01			
IIL-8			0.04	
*IIIL-5*			1.00	
*IIIL-34*				1.00
IIIL-47		0.75		
IIIL-48		0.02		
*IIIL-49*				1.00
IIIL-50				0.72
IIIL-51				0.78
IIIL-52				0.67
IIIL-53				0.11
*IIIL-54*			1.00	
*IIIL-55*			1.00	
IIIL-56			0.08	

^1^ CR = Chromosomal rearrangements (other than fixed sequences shared by all known members of the *S*. *tani* complex); an inversion is italicized if it is fixed in all cytoforms in which it is present.

#### *Simulium tani* Cytoform ‘B2’

We analyzed the banding patterns of all 48 chromosome preparations of larvae from Site 5. Fixed inversions were absent. IL-2 was absent, in contrast to its preponderance in ‘B’ in Thailand [19]; on this basis, we recognized 2 subunits of Cytoform ‘B’: ‘B1’ in Thailand and ‘B2’ in Vietnam. The sex chromosomes were undifferentiated. Polymorphisms were scarce; 1 male and 1 female were heterozygous for IS-25 (Fig 1) and IL-15 (Fig 3), respectively (Table 3).

#### *Simulium tani* Cytoform ‘M’

Material of this cytoform was identified as a distinct morphotaxon, ‘b’, of *S*. *tani*. The chromosomal banding patterns of 22 larvae (Site 1) were analyzed entirely. Larvae were fixed for *IL-2* and carried IIIL-47 (Fig 5) in high frequency (0.75), without significant linkage to gender (females: 1 ss, 1 si, 6 ii; males: 1 ss, 7 si, 6 ii; where s = standard sequence, i = inverted sequence; χ^2^ = 0.15, df = 1, P > 0.05), but in Hardy-Weinberg equilibrium (χ^2^ = 0.265, df = 1, P > 0.05). The only other rearrangements were IS-22 (Fig 2A) and IIIL-48 (Fig 5), which occurred heterozygously in 1 male and 1 female larva, respectively (Table 3). All 3 polymorphisms were unique to Cytoform ‘M’.

#### *Simulium tani* Cytoform ‘N’

Twelve larvae of this cytoform were discovered in the same collection (Site 5) as the 48 larvae of Cytoform ‘B2’; all 60 larvae collectively were referred to as morphoform ‘a’. Cytoform ‘N’ was characterized by fixed inversions *IIIL-5*, *IIIL-54*, and *IIIL-55* (Fig 6) and a high frequency (0.92) of IL-14 (Table 3; Fig 3). Three additional inversions were found heterozygously in low frequency, all except IIL-8 (Fig 4B) in male larvae. No inversion was linked conclusively to gender in our small sample. All inversions were unique to Cytoform ‘N’ except IL-2 (frequency = 0.08) and *IIIL-5*, which are found in numerous cytoforms of the *S*. *tani* lineage. No hybrids were found between ‘B2’ and ‘N’, indicating that they were reproductively isolated.

#### *Simulium xuandei* Takaoka & Pham

The banding patterns of all 9 prepared larvae from Site 4 were analyzed completely (Table 3). Compared with the typical banding sequence of *S*. *tani*, all larvae were fixed for *IS-23* (Fig 1B), *IL-2*, *IIIL-34*, and *IIIL-49* (Fig 7). Polymorphic inversions IIIL-50, IIIL-51, and IIIL-52 (Fig 7) were present in two-thirds or more of all 18 homologues, whereas IS-24 (Fig 1B) and IIIL-53 (Fig 7) occurred in only 1 or 2 homologues, respectively. Sex chromosomes were microscopically undifferentiated.

### Southeast Asian *Simulium tuberosum* subgroup

**Fig 8:**
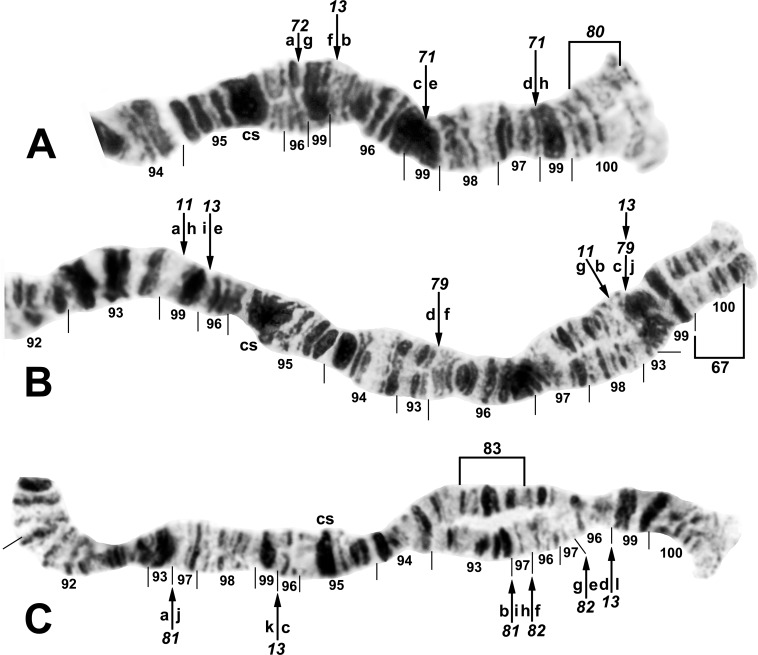
Distal portion of IIIL arm of *Simulium tuberosum* group from Vietnam. The sequence of the *Simulium* subgeneric standard [19] can be obtained by alphabetically ordering the fragments indicated by small letters (plus inverting *IIIL-80* for *S*. *congi*); cs = cup and saucer marker. A. *Simulium congi* (female larva, Site 3), showing the *IIIL-13*,*71*,*72*,*80* sequence. The sequence involves 2 coincident breakpoints. Hence, inverting *IIIL-71* will create the e/h junction representing the distal break of *IIIL-72*; when *IIIL-72* is then inverted, it creates the a/e junction representing the proximal break of *IIIL-13*. B. *Simulium doipuiense* ‘C’ (female larva, Site 3), showing the *IIIL-11*,*13*,*79* sequence; section 100, however, is of *Simulium doipuiense* ‘D’ (female larvae, Site 6), showing heterozygous expression of IIIL-67. C. *Simulium brevipar* ‘B’ (male larva, Site 9), showing the *IIIL-13*,*81*,*82* sequence and heterozygous expression of IIIL-83.

**Fig 9:**
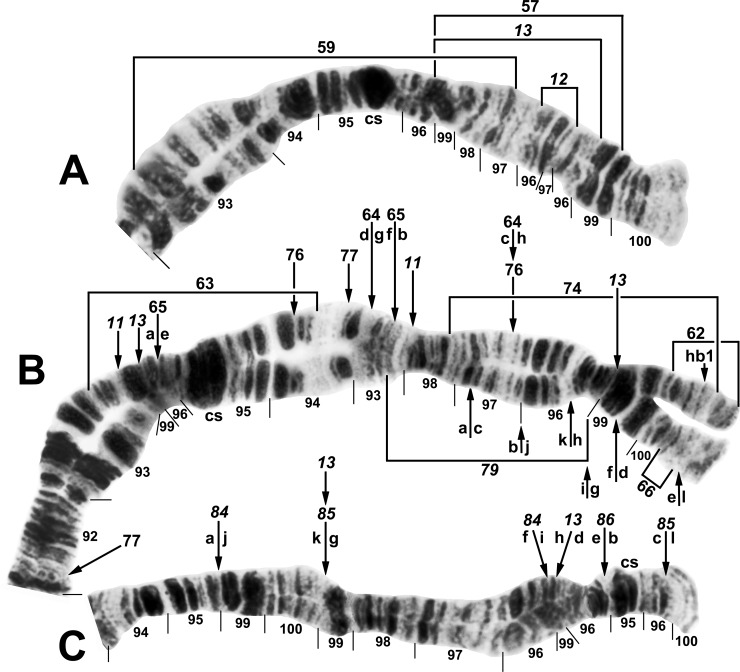
Distal portion of chromosome IIIL (female larvae) of *Simulium tuberosum* group from Vietnam. A. *Simulium yuphae* ‘A’ (Site 6), showing the *IIIL-12*,*13* sequence. Breakpoints of polymorphic inversions IIIL-57 and IIIL-59 of *S*. *yuphae* ‘B’ and ‘A’, respectively, are indicated by brackets; cs = cup and saucer marker. B. *Simulium doipuiense* ‘A’ (Site 15), showing the *IIIL-11*,*13* sequence. Polymorphic inversion IIIL-65 occurs on top of IIIL-64; the inverted sequence for these 2 inversions can be obtained by alphabetizing the letters a–h. A complex set of distal inversions (Fig 10) can be obtained on top of the *IIIL-11*,*13* sequence by alphabetizing the fragments indicated by the letters a–1, corresponding to hypothetical inversions IIIL-90,91,92,93,94 (not individually identified); hb = location of heteroband. C. *Simulium brevipar* ‘C’ (Site 12), showing the *IIIL-13*,*84*,*85*,*86* sequence. Inverting *IIIL-84* will create the a/e breakpoint for *IIIL-86*. The sequence of the *Simulium* subgeneric standard [19] can be obtained by alphabetically ordering the fragments indicated by the letters a–1.

**Fig 10:**
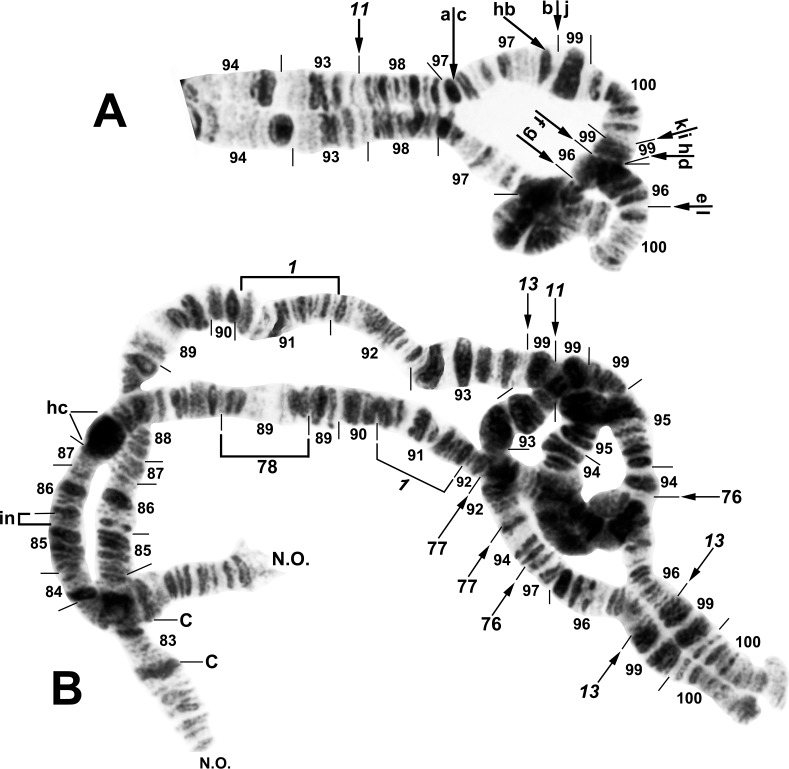
Complex heterozygous inversions in IIIL of *Simulium doipuiense* ‘A’ (Site 15) from Vietnam. A. Female larva showing 1 of several possible hypotheses for band identities and breakpoints; based on this hypothesis, the *IIIL-11*,*13* sequence can be obtained by alphabetically ordering the fragments indicated by the letters a–1, corresponding to hypothetical inversions IIIL-90,91,92,93,94 (not individually identified). B. Male larva showing the *IIIL-11*,*13* sequence with IIIL-76,77,78, plus 85i and 87/88hc, on 1 homologue; *IIIL-1* of the entire *S*. *tuberosum* group is present (bracketed).

**Fig 11:**
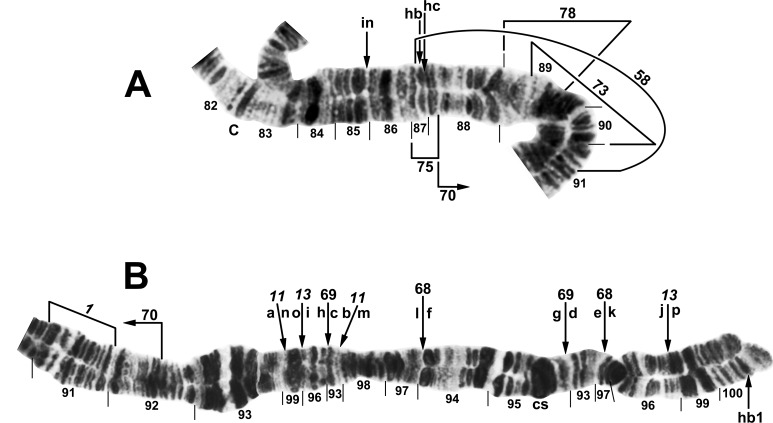
Chromosome IIIL (female larvae) of the *Simulium tuberosum* group from Vietnam. *IIIL-1* is present (i.e., section 91 is inverted). A. Base of *S*. *brevipar* ‘C’ (Site 12). Limits of polymorphic inversions IIIL-58 of *S*. *yuphae* ‘B’ and IIIL-70, IIIL-73, IIIL-75, and IIIL-78 of the *S*. *doipuiense* complex are indicated by brackets. C = centromere; hb = location of heteroband of *S*. *rufibasis* ‘B’; hc and in = locations of heterochromatic block and of 2 fine band inserts, respectively, of *S*. *doipuiense* ‘A’. B. Distal portion of *Simulium doipuiense* ‘E’ (Site 15), showing the probable X-linked IIIL-68,69 sequence on top of *IIIL-11*,*13*, plus homozygous mild expression of heteroband 100hb1. The *Simulium* subgeneric standard sequence [19] can be obtained by alphabetically ordering the fragments indicated by the letters a–p; cs = cup and saucer marker.

**Fig 12:**
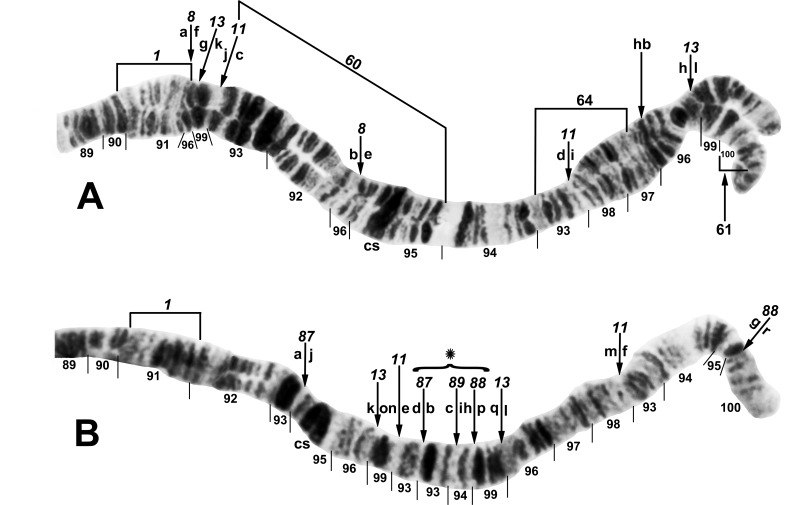
Distal portion of chromosome IIIL of the *Simulium tuberosum* group from Vietnam. *IIIL-1* is present (i.e., section 91 is inverted). The sequence of the *Simulium* subgeneric standard [19] can be obtained by alphabetically ordering the fragments indicated by small letters; cs = cup and saucer marker. A. *Simulium rufibasis* ‘B’ (male larva, Site 12), showing the *IIIL-8*,*11*,*13* sequence; subterminal inversion IIIL-61 is expressed heterozygously, and breakpoints of polymorphic inversions IIIL-60 and IIIL-64 are indicated by brackets; hb = location of heteroband. B. *Simulium doipuiense* ‘F’ (female larva, Site 15), showing the *IIIL-11*,*13*,*87*,*88*,*89* sequence; the second breakpoint for *IIIL-89* follows the inversion of *IIIL-87*, creating the d/j junction. Tentative breakpoints and section numbers are indicated with a bracket and asterisk (*) above them.

We recognized 11 cytoforms among 6 morphoforms in the Southeast Asian *S*. *tuberosum* subgroup in Vietnam (Table 1). Relative to the *Simulium* subgeneric standard sequence, all Vietnamese members of this subgroup shared *IL-1*, *IL-tuberosum*, 4 inversions in IIS, 3 inversions in IIL, and *IIIL-1* [19]; these inversions are not repeated in the descriptions below or in Table 4. Figs 1, 2, 4, and 8–12 depict all rearrangements discovered in the subgroup.

**Table 4:** Frequency of chromosomal homologues with rearrangements in the Southeast Asian *Simulium tuberosum* subgroup in Vietnam.

CR^1^	Cytoform (*n*)											
	*brevipar* A^2^ (8)	*brevipar* B (2)	*brevipar* C (1)	*congi* (4)	*doip*.^3^ A (113)	*doip*. C (1)	*doip*. D (14)	*doip*. E (8)	*doip*. F (1)	*rufibasis* B (14)	*yuphae* A (21)	*yuphae* B (2)
IS-26	0.69											
IS-27										0.04*^4^		
IS-28										0.14*		
IS-29										0.25*		
IS-30					0.004							
IS-31										0.25*		
*IS-32*						1.00						
IS 13hb										0.25*		
IS rptn^5^					0.004							
*IL-16*						1.00						
IL-17					0.004							
IL-18					0.004							
IL-19					0.004			0.06				
IL 24hc							0.07					
IIS-7					0.009							
IIS-8					0.004							
IIS-9					0.004							
IIS-10					0.004							
IIS 42hc					0.004							
*IIIL-8*										1.00		
*IIIL-11*					1.00	1.00	1.00	1.00	1.00	1.00		
IIIL-12										0.36	1.00	1.00
*IIIL-13*	1.00	1.00	1.00	1.00	1.00	1.00	1.00	1.00	1.00	1.00	1.00	1.00
IIIL-57												0.25*
IIIL-58												0.50*
IIIL-59											0.07	
IIIL-60										0.39		
IIIL-61										0.04		
IIIL-62					0.022							
IIIL-63					0.004							
IIIL-64					0.009					0.04		
IIIL-65					0.004							
IIIL-66					0.009							
IIIL-67							0.07					
IIIL-68								0.88*				
IIIL-69								0.75*				
IIIL-70								0.06*				
*IIIL-71*				1.00								
*IIIL-72*				1.00								
IIIL-73					0.009							
IIIL-74					0.004							
IIIL-75					0.004							
IIIL-76					0.009							
IIIL-77					0.009							
IIIL-78					0.004							
*IIIL-79*						1.00	1.00					
*IIIL-80*				1.00								
*IIIL-81*		1.00										
*IIIL-82*		1.00										
IIIL-83		0.50*										
*IIIL-84*			1.00									
*IIIL-85*			1.00									
*IIIL-86*			1.00									
*IIIL-87*									1.00			
*IIIL-88*									1.00			
*IIIL-89*									1.00			
IIIL-90cplx^6^					0.013							
IIIL 85in					0.004							
IIIL 87hb										0.04		
IIIL 87/88hc					0.009							
IIIL 96hb										0.04		
IIIL 97hb					0.013							
IIIL 100hb1					0.009			0.88*				
IIIL 100hb2^7^					0.004							
IIIL 100hc^8^					0.004							

^1^ CR = Chromosomal rearrangements (other than fixed sequences shared by all known members of the Southeast Asian *S*. *tuberosum* subgroup); an inversion is italicized if it is fixed in all cytoforms in which it is present. Sites were combined within each cytoform.

^2^ *Simulium brevipar sensu stricto* (= Cytoform ‘A’) was found only in Malaysian samples (Sites 17, 18).

^3^ *doip*. = *Simulium doipuiense*.

^4^ * = Implicated as sex linked.

^5^ A segment of IS (Fig 1) was heterozygously repatterned (rptn), with most bands differentially expressed.

^6^ IIIL-90cplx represents as many as 5 inversions (IIIL-90,91,92,93,94) relative to the *IIIL-11*,*13* sequence. The bands and breakpoints are not all sufficiently homologized to confidently resolve all inversions; 1 possible hypothesis is presented in Figs 9B and 10.

^7^ The telomere of 1 homologue was represented by a heteroband in a male larva from Site 13.

^8^ The telomere of 1 homologue was represented by a heterochromatic block in a female larva from Site 12.

#### *Simulium brevipar* Cytoform ‘A’

To establish the chromosomal characteristics of *S*. *brevipar*, we examined 8 larvae from the Cameron Highlands of Malaysia (Sites 17, 18) about 90 km from the type locality. Accepting these larvae as chromosomally representative of the type of *S*. *brevipar*, and assigning them to Cytoform ‘A’, we found that our 2 samples, albeit small, were cohesive. Larvae were fixed for *IIIL-13*. The only polymorphism (autosomal) was IS-26 (Fig 2A), homozygous in 4 males, heterozygous in 2 females and 1 male, and standard in 1 female (Table 4). The small sample revealed no evidence of differentiated sex chromosomes.

#### *Simulium brevipar* Cytoform ‘B’

Two male larvae in a sample (Site 9) identified morphologically as *S*. *brevipar*, based on characters of associated pupae and adult males (e.g., number of upper-eye facets), had a unique sequence in IIIL, with inversions *IIIL-13*, *IIIL-81*, and *IIIL-82* (Table 4; Fig 8C). Both larvae were heterozygous for IIIL-83 (Fig 8C), suggesting possible Y-chromosome linkage. We tentatively regard these larvae as Cytoform ‘B’, distinct from *S*. *brevipar sensu stricto* (i.e., ‘A’).

#### *Simulium brevipar* Cytoform ‘C’

The sole larva (female, Site 12) of this cytoform was homozygous for 3 unique inversions—*IIIL-84*, *IIIL-85*, and *IIIL-86*—on top of *IIIL-13* (Table 4; Fig 9C). Although we show the 3 unique inversions as fixed (italicized), larger samples are needed for confirmation. No other rearrangements were present. The only basis for assigning this cytoform to the *S*. *brevipar* complex was the presence of *IIIL-13* without *IIIL-11* or IIIL-12.

#### *Simulium congi* Takaoka & Sofian-Azirun

A small sample of 4 larvae from Sites 2 and 3, including the type locality, was analyzed completely. IIIL had 4 fixed inversions: *IIIL-13*, *IIIL-71*, *IIIL-72*, and *IIIL-80* (Table 4; Fig 8A). We acknowledge that some of these inversions might be polymorphic in larger samples. Sex chromosomes were undifferentiated and polymorphisms were absent.

#### *Simulium doipuiense* Cytoform ‘A’

The 113 larvae of this cytoform from Sites 11–16 in northern Vietnam conformed to the basic sequence of *S*. *doipuiense* ‘A’ [19], *viz*. fixation of *IIIL-11* and *IIIL-13* (Fig 9B), with cytologically undifferentiated sex chromosomes (or predominantly undifferentiated if rearrangements, for example, in the terminus of IIIL, such as IIIL-62 in 5 males and IIIL-66 in 2 males, are Y linked). Although the 32 different polymorphisms were each expressed in low frequency (< 0.03; Table 4), complex linkage groups were disproportionately represented, with no apparent relation to gender. At Site 15, for example, 10 of 52 larvae carried all 35 (22 different) polymorphisms; 5 of these larvae expressed 30 of the polymorphisms (18 different): 2 females and 1 male had IIIL-90cplx (hypothesized to consist of inversions IIIL-90,91,92,93,94) plus 97hb on 1 homologue (Figs 9B and 10A), and 1 of these females also had IIS-8,9+42hc on 1 homologue, while the male was heterozygous for IIS-10 (Fig 4A); a second male had 1 homologue with IIIL-76,77,78 plus 2 fine band insertions (85i) and a large heterochromatic block (87/88hc) in the base of the arm (Figs 9B and 10B), whereas a third male carried IIIL-73,74,75 on 1 homologue (Figs 9B and 11A). Additional rare rearrangements included IS-30 (Fig 1A), IL-17, IL-18, IL-19 (Fig 2C), and others in IIIL (Fig 9B).

#### *Simulium doipuiense* Cytoform ‘C’

We analyzed 1 larva (female, Site 3) of this species, mixed with a sample identified morphologically as *S*. *congi*. *IIIL-11* and *IIIL-13* were present. *IS-32*, *IL-16*, and *IIIL-79* also were homozygous (Table 4; Figs 1A, 2B, and 8B), but whether they actually were fixed, as provisionally represented, could not be determined without larger samples. Although only a single larva was found, we tentatively recognized it as a separate cytoform on the strength of homozygous inversions *IS-32*, *IL-16*, and *IIIL-79*.

#### *Simulium doipuiense* Cytoform ‘D’

The 14 available larvae (Sites 6, 7) of *S*. *doipuiense* ‘D’, similar to the single larva of ‘C’ from Site 3, were fixed for *IIIL-11*, *IIIL-13*, and *IIIL-79* (Table 4), but lacked *IS-32* and *IL-16*. One female larva was heterozygous for IIIL-67 (Fig 8B), which differed from IIIL-62 of cytoform ‘A’ (Fig 9B) by only 1 band at each end. One male was heterozygous for a large heterochromatic insert in section 24 of IL (Fig 2C). The sex chromosomes were microscopically undifferentiated (X_0_Y_0_). Larvae occupied the same streams as *S*. *yuphae* ‘A’, and the two cytoforms were reproductively isolated.

#### *Simulium doipuiense* Cytoform ‘E’

This cytoform carried the typical *IIIL-11*,*13* sequence of the *S*. *doipuiense* and *S*. *rufibasis* complexes (Table 4). It was uniquely characterized by probable X linkage of rearrangements (Table 5; Fig 11): X_1_ (IIIL-68 plus mildly enhanced heteroband 100hb1), further elaborated as X_2_ by the overlay of inversion IIIL-69 (IIIL-68,69+100hb1) and rarely as X_3_ (IIIL-68,69,70+100hb1). Accepting IIIL-68 as X linked, implied that the Y chromosome was standard (Y_0_; i.e., had no rearrangements other than the *IIIL-11*,*13* sequence). The only autosomal polymorphism was IL-19 (Fig 2C), heterozygous in 1 female. *Simulium doipuiense* ‘E’ was collected from the same stream (Site 15) with ‘A’. If IIIL-68 was X linked in ‘E’, the presence of 25 females standard for this inversion and 6 females inverted for it, with no heterozygotes, argues for reproductive isolation of ‘A’ and ‘E’. The only shared rearrangements between ‘A’ and ‘E’, other than *IIIL-11*,*13* were IL-19 and the putatively X-linked IIIL 100hb1.

**Table 5:** Distribution by gender of possible sex-linked rearrangements in IIIL of *Simulium doipuiense* ‘E’, Vietnam.

Gender	Sex-chromosome classes^1^			
	X_1_ X_2_	X_2_ X_2_	X_2_ X_3_	X_2_ Y_0_
Female	2	3	1	0
Male	0	0	0	2

^1^ All classes carry the *IIIL-11*,*13* sequence. X_1_ = IIIL-68+100hb1, X_2_ = IIIL-68,69+100hb1, X_3_ = IIIL-68,69,70+100hb1, and Y_0_ = standard sequence (i.e., only *IIIL-11*,*13*).

#### *Simulium doipuiense* Cytoform ‘F’

The single larva (female, Site 15) of this cytoform had a novel IIIL banding pattern with 3 homozygous inversions—*IIIL-87*, *IIIL-88*, and *IIIL-89*—on top of *IIIL-11*,*13* (Table 4; Fig 12B). We provisionally show *IIIL-87*,*88*,*89* as fixed (italicized), pending more material. Our interpretation of bands in sections 93/93/94, and consequently the included breakpoints, is tentative. The banding sequence of the remainder of the polytene complement conformed to that of the Southeast Asian *S*. *tuberosum* subgroup. The larva was coinfected with 2 unknown species of microsporidia.

#### *Simulium rufibasis* Cytoform ‘B’

All 14 larvae of this species from Sites 11–13 were analyzed completely. *IIIL-8*, *IIIL-11*, and *IIIL-13* were fixed (Table 4; Fig 12A). IIIL-12 (Fig 9A), previously known only as a fixed inversion in *S*. *yuphae* and *S*. ‘unknown sp. 2’ of Tangkawanit et al. [19], was an autosomal polymorphism at Site 12. IIIL-60 was a common autosomal polymorphism, and IIIL-61 was heterozygous in 1 male larva (Fig 12A). Two heterobands in IIIL (87hb and 96hb) were found as single heterozygotes (Figs 11A and 12A). IIS-4, an inversion originally interpreted as present in *S*. *rufibasis* [19] was absent in our larvae; however, the symmetry of bands within this small inversion in some preparations suggests that the original [19] interpretation of the presence of this inversion in *S*. *rufibasis* might have been erroneous. IIIL-64 (Fig 9B), which appeared heterozygously in the only larva (female) of the *S*. *rufibasis* complex at Site 11, was shared with *S*. *doipuiense* ‘A’ at the same site. The presence of IS-29,31+13hb (= Y_1_) in 7 of 9 males but in none of the 3 females at Site 12 suggests that IS is the sex arm (Table 6; Fig 1A). IS-27 (X_1_) and IS-28 (X_2_) might represent alternative X sequences to the undifferentiated X_0_; males also were polymorphic for an undifferentiated Y_0_ chromosome (Table 6; Fig 1A). We tentatively recognize our Vietnamese material as a new cytoform, ‘B’, on the basis of probable differentiated sex chromosomes, in contrast to material of *S*. *rufibasis* with cytologically undifferentiated sex chromosomes, previously analyzed from Thailand [19], and recognized here, retrospectively, as Cytoform ‘A’. *Simulium rufibasis* ‘B’ and *S*. *doipuiense* ‘A’ occurred in the same streams and were reproductively isolated from one another.

**Table 6:** Distribution by gender of possible sex-linked rearrangements of *Simulium rufibasis* ‘B’, Vietnam (Site 12).

Gender	Sex-chromosome classes^1^						
	X_0_X_0_	X_0_X_2_	X_0_Y_0_	X_0_Y_1_	X_1_Y_0_	X_2_Y_0_^2^	X_2_Y_1_
Female	1	1	0	0	0	0	0
Male	0	0	1	4	1	1	2

^1^ Assuming IS-29,31+13hb is Y-linked, X_0_ and Y_0_ = standard sequence, X_1_ = IS-27, X_2_ = IS-28, and Y_1_ = IS-29,31+13hb. The only female at Site 11 and the only male at Site 13 were X_0_X_0_ and X_0_Y_0_, respectively; they are not included in the table.

^2^ 1 additional larva of undetermined gender (infected with a microsporidium) was heterozygous for IS-28.

#### *Simulium yuphae* Cytoform ‘A’

We analyzed 21 larvae from Sites 6–9. The larvae, identified morphologically as *S*. *cavum* Takaoka & Ya’cob, were chromosomally classic for *S*. *yuphae*, having *IIIL-12* and *IIIL-13* (Fig 9A), with undifferentiated sex chromosomes and few polymorphisms. One male larva from Site 6 and 1 female from Site 7 had the typical sequence for *S*. *yuphae* but were heterozygous and homozygous, respectively, for IIIL-59 (Table 4; Fig 9A). We tentatively consider IIIL-59 an autosomal polymorphism of *S*. *yuphae* ‘A’, although the possibility that it is X linked (and possibly associated with a separate breeding population) cannot be excluded.

#### *Simulium yuphae* Cytoform ‘B’

Two male larvae (Site 10), initially segregated as morphologically distinct, were chromosomally identical to *S*. *yuphae* ‘A’, with *IIIL-12* and *IIIL-13*, except both were heterozygous for IIIL-58 (Table 4; Fig 11A) and 1 also was heterozygous for IIIL-57 (Fig 9A), suggesting possible sex linkage of the 2 inversions. We, therefore, provisionally regard them as a separate cytoform—‘B’. No other rearrangements were present. If ‘B’ is consistently defined by a differentiated Y chromosome, then the 2 females from Site 9, about 215 km away, also could belong to ‘B’.

### Phylogenetic Relationships

Rearrangements previously identified as synapomorphies for the *S*. *tuberosum* group, *S*. *tani* complex, and Southeast Asian subgroup [24] were included in our phylogenies. Among the 88 chromosomal rearrangements discovered in the *S*. *tuberosum* group in Vietnam, 8 had phylogenetic potential (*IL-2*, IL-19, *IIIL-11*, IIIL-12, *IIIL-13*, IIIL-64, *IIIL-79*, and 100hb1); that is, they were shared between at least 2 taxa. Seven of these rearrangements were uniquely derived (synapomorphic), based on outgroup comparisons; the breakpoints of IL-19 (shared by *S*. *doipuiense* ‘A’ and ‘E’) could not be determined in the outgroups and, therefore, was not used for phylogenetic inference. The probability that a shared heteroband (e.g., 100hb1) represents common ancestry versus independent origins is not known. However, the likelihood of independently enhancing DNA content of a band is probably greater than independently sharing an inversion with two microscopically identical breakpoints; thus, we consider the phylogenetic value of 100hb1 weak.

**Fig 13:**
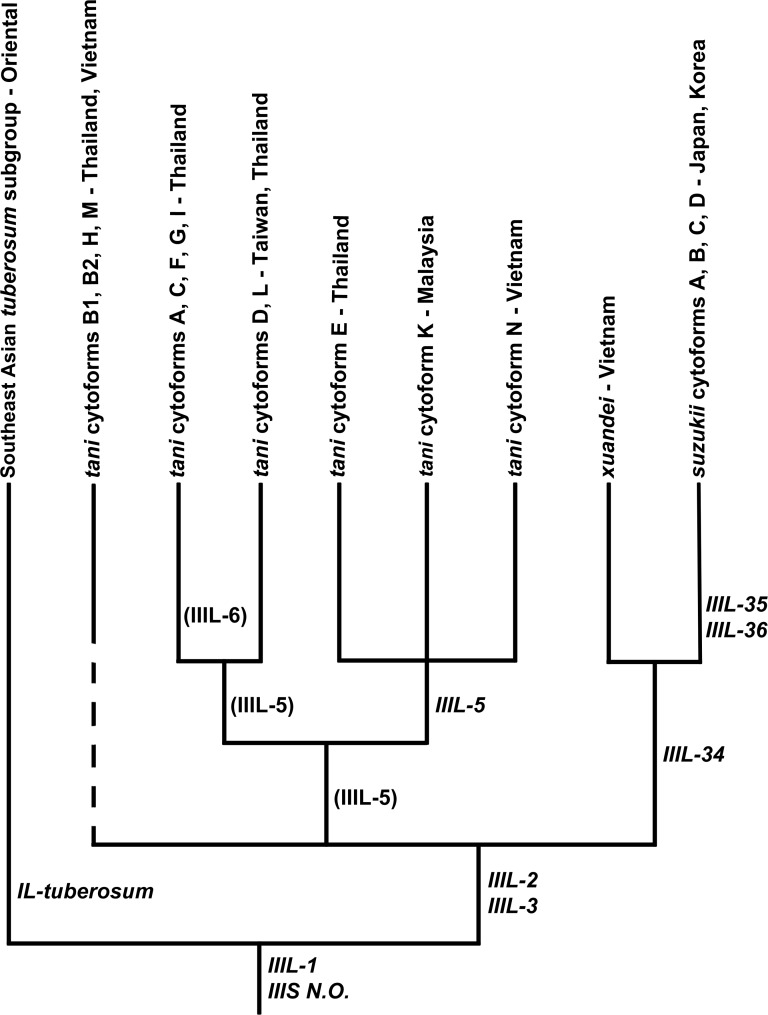
Cytophylogeny of the *Simulium tani* complex. The outgroups (*Simulium erythrocephalum* and *Simulium vittatum*) are not shown. Italicized rearrangements are fixed; polymorphisms are in parentheses. The dashed line for *S*. *tani* B1, B2, H, and M indicates that these taxa have no defining rearrangements. The 4 cytoforms of the northern *S*. *suzukii* lineage have been resolved previously [24], and the Southeast Asian *S*. *tuberosum* subgroup is resolved in Fig 14.

Within the *S*. *tani* lineage, *S*. *xuandei* was the sister species of the northern clade of *S*. *suzukii*, based on *IIIL-34*, whereas *S*. *tani* ‘B2’ and ‘M’, lacking both IL-2 and IIIL-5, were in an unresolved trichotomy with all other members of the *S*. *tani* complex (Fig 13). *Simulium tani* ‘N’ was in an unresolved trichotomy with cytoforms ‘E’ and ‘K’.

**Fig 14:**
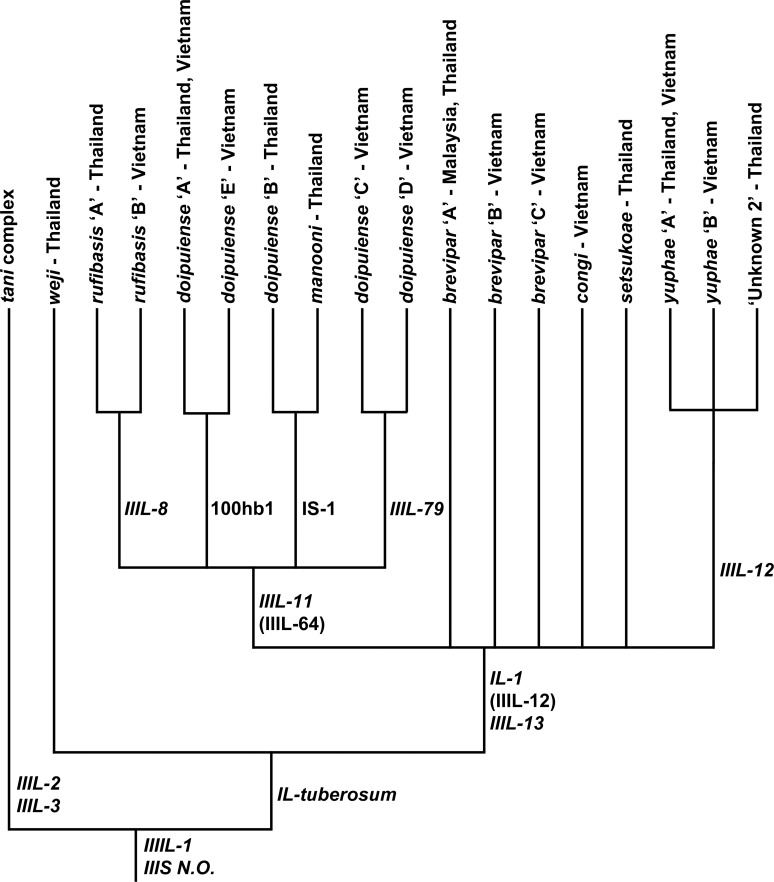
Cytophylogeny of the Southeast Asian *Simulium tuberosum* subgroup. Rearrangements are used only if they were determined to be derived relative to the outgroups (*Simulium erythrocephalum* and *Simulium vittatum*; not shown). Italicized rearrangements are fixed; polymorphisms are in parentheses.

The Southeast Asian *S*. *tuberosum* subgroup was uniquely defined by fixed inversion *IL-tuberosum* (Fig 14). Within the *IIIL-13* clade, one lineage included the *IIIL-11* clade consisting of the cytoforms of *S*. *rufibasis*, defined by *IIIL-8*, and the cytoforms of *S*. *doipuiense*, whereas another lineage, defined by fixation of *IIIL-12*, included the *S*. *yuphae* cytoforms. IIIL-12 is shown as a polymorphism in the ancestor of the *S*. *doipuiense-rufibasis-yuphae* lineage to accommodate its presence as a polymorphism in *S*. *rufibasis* ‘B’ and as a fixed inversion in the *S*. *yuphae* line; under this hypothesis, IIIL-12 would have been lost (or not yet discovered) in all other members of the *IIIL-13* clade. *Simulium congi* and the cytoforms of *S*. *brevipar* were in an unresolved polytomy in the *IIIL-13* clade. IIIL-64 is shown as a polymorphism in the ancestor of the *S*. *doipuiense-rufibasis* clade to account for its presence in *S*. *doipuiense* ‘A’ and *S*. *rufibasis* ‘B’, although the alternative hypothesis is that it was introduced into one or the other by introgression, having been found in both cytoforms at the same site.

## Discussion

### Taxonomic status of cytoforms

We discovered 15 cytoforms among 9 morphoforms of 6 nominal species in the *S*. *tuberosum* group in Vietnam. The cytoforms fall into two categories based on the evidence that can be mustered for reproductive isolation: (1) valid (i.e., reproductively isolated) species and (2) taxa with insufficient information for determining species status. Compelling evidence exists to recognize five cytoforms as reproductively isolated from all other members of the group: *Simulium tani* ‘B2’ (or ‘N’), *S*. *tani* ‘M’, *S*. *xuandei*, *S*. *congi*, and *S*. *doipuiense* ‘A’. Cytoforms whose taxonomic status cannot be assessed because of lack of sympatry, absence of fixed chromosomal differences, lack of morphological or molecular evidence, or a limited sample size—all of which can preclude detection of hybrids—include *S*. *tani* ‘N’, *S*. *brevipar* ‘B’, *S*. *brevipar* ‘C’, *S*. *doipuiense* ‘C’, *S*. *doipuiense* ‘D’, *S*. *doipuiense* ‘E’, *S*. *doipuiense* ‘F’, *S*. *rufibasis* ‘B’, *S*. *yuphae* ‘A’, and *S*. *yuphae* ‘B’.

*Simulium tani* ‘B’ is precariously defined by a lack of diagnostic chromosomal rearrangements relative to all known members of the *S*. *tani* complex [19,24,30]. Two points merit discussion: (1) Our material of Cytoform ‘B2’ in Vietnam conforms chromosomally to ‘B1’ in Thailand [19], except IL-2 is absent in Vietnam, compared to its typically high frequency in Thailand where 9 of 16 samples had IL-2 frequencies of 1.00. This discrepancy is reconciled if ‘B1’ in Thailand actually includes two species, one lacking IL-2 and another fixed or polymorphic for IL-2. One large Thai population (Site 41) that was not in Hardy-Weinberg equilibrium had a dearth of IL-2 heterozygotes [19], supporting the idea of two species within ‘B’. (2) Molecular and morphological analyses, including material from our Site 5 in Vietnam and from Site 37 in Thailand (ca. 680 km distant), previously recorded [19] as fixed for IL-2, indicate that Vietnamese ‘B2’ (= *S*. *tani* ‘a’) and Thai ‘B1’ are distinct species and that Vietnamese ‘B2’ is distinct from all other analyzed populations of the *S*. *tuberosum* group [13]. Complicating this analysis, however, is the chromosomal evidence—three fixed-inversion differences—for the existence of a second, separate breeding population (*S*. *tani* ‘N’) in the same stream with ‘B2’. The molecular analyses [13], however, did not discern two species; therefore, we do not know if the single species recognized molecularly was ‘B2’ or ‘N’ (or both), although ‘B2’ had greater representation (80%) in our chromosomal sample.

*Simulium tani* ‘M’ (= *S*. *tani* ‘b’) has molecular and morphological support as a distinct species [13], but only moderate chromosomal support—a high frequency (0.75) of the unique inversion IIIL-47. *Simulium xuandei*, on the other hand, has strong molecular, morphological [13], and chromosomal support for species status. Yet, molecular analyses of material of *S*. *xuandei* collected simultaneously with our chromosomal sample revealed two or three cryptic species [13], whereas our chromosomal sample recovered only a single species. With a larger chromosomal sample, the molecular hypothesis of separate breeding populations could be tested.

Our Malaysian sample of *S*. *brevipar sensu stricto* (= ‘A’) from near the type locality had a fixed banding sequence identical to that of larvae collected with *S*. *yuphae* in Thailand (Site 59) by Tangkawanit et al. [19] and recognized by those authors as a probable species distinct from *S*. *yuphae*. The chromosomal analysis of our Malaysian samples of *S*. *brevipar*, thus, retrospectively confirms the presence of *S*. *brevipar* ‘A’ in Thailand. Specimens in Vietnam designated *S*. *brevipar* ‘B’ differ from *S*. *brevipar* ‘A’ by two putatively fixed inversions. Our unpublished analysis of the cytochrome *c* oxidase subunit II (COII) gene indicates minimal (0.2%) genetic distance within *S*. *brevipar* ‘A’ versus 1.4–1.5% between ‘A’ and ‘B’, lending support to the possibility that they are separate species. However, the alternative possibility that ‘B’ is conspecific with ‘A’ cannot be excluded; more than 1900 linear km separate our samples. The Nearctic example of *S*. *congareenarum* provides a caveat. Two populations of *S*. *congareenarum* more than 1200 km apart originally were proposed as sibling species based on two fixed-inversion differences supported by slight morphological differences; eventual analysis of an intervening population revealed heterozygosity for the two inversions, reflecting polymorphism gradients and, thus, a single species with fixation of alternate sequences at the two geographic sampling extremes [31]. Geographically intermediate collections and larger samples are needed to test reproductive isolation of *S*. *brevipar* ‘A’ and ‘B’.

Three unique chromosomal inversions and the novel swollen basal fenestra of the pupal gill of *S*. *congi* [18] support its species status. *Simulium brevipar* ‘C’ and *S*. *doipuiense* ‘F’, however, are enigmatic victims of inadequate sample sizes, and little can be said about their status. Nonetheless, the three unique inversions in each cytoform have a low probability of being polymorphisms that happen to be expressed homozygously in single larvae. The microsporidia-infected larva of ‘F’ might have been a remnant of a larger population of chromosomally similar larvae that already had developed, leaving only parasitized individuals. Parasitized larvae typically persist in populations after unparasitized larvae have pupated [32].

*Simulium doipuiense sensu stricto* (= ‘A’) has a consistent fixed banding sequence and undifferentiated sex chromosomes over its known range, including sites within 4 km of the type locality [19]. None of its polymorphisms, however, are shared between populations in Thailand and Vietnam. Chromosomal evidence demonstrates that *S*. *doipuiense* ‘A’ is reproductively isolated from the two members (‘A’ and ‘B’) of the *S*. *rufibasis* complex; no hybrids have been found in sympatry in Thailand [19] or in our study. The presence of IIIL-64 in one larva of *S*. *rufibasis* ‘B’ and in two larvae of *S*. *doipuiense* ‘A’ from the same site suggests either introgression or retention of an ancestral polymorphism. Molecular analyses fail to distinguish the *S*. *doipuiense* and *S*. *rufibasis* complexes in Thailand [33,34]. Ecologically, the *S*. *rufibasis* complex inhabits higher elevations (1100–2300 m) than does *S*. *doipuiense* ‘A’ (400–1800 m) [19].

Although *S*. *doipuiense* ‘D’ in central Vietnam differs from ‘A’ by fixation of *IIIL-79*, our samples of the two cytoforms are separated by about 800 linear km. The scenario represents another example of the difficulty of interpreting the extent of reproductive isolation between distant populations. The case for *S*. *doipuiense* ‘C’ (previously referred to as *S*. *rufibasis* [18]), collected about 450 km to the south of ‘D’, is similar, but is further confounded by a sample of only one larva. Geographically intermediate collections are needed to test reproductive isolation of ‘A’, ‘C’, and ‘D’. *Simulium doipuiense* ‘E’, collected from the same stream with ‘A’, is tentatively regarded as a distinct species, based on an absence of hybrids. Greater confidence in claiming reproductive isolation would come from a larger sample or molecular or morphological corroboration.

*Simulium rufibasis* ‘B’ differs from its nearest relative, *S*. *rufibasis sensu stricto* (= ‘A’), only in its putative sex chromosomes and autosomal polymorphisms. ‘B’ might be merely an example of sex-chromosome polymorphism, which is common in the Simuliidae [11], but we treat it here as a cytoform in recognition of the asserted role that sex chromosomes play in speciation of Diptera [35], including the Simuliidae [36,37]. To provide a taxonomic anchor, we provisionally regard ‘A’ as conspecific with chromosomally unstudied topotypical material of *S*. *rufibasis* from India. In the absence of molecular and morphological data, an assessment of the taxonomic status of ‘B’ relative to ‘A’ is precluded.

*Simulium yuphae* ‘A’ in our samples was identified morphologically as *S*. *cavum*. However, we found no chromosomal differences between Thai populations of ‘A’, including topotypical material of *S*. *yuphae* [19], and our material of *S*. *yuphae* (morphologically *S*. *cavum*) collected about 750–900 km to the east. Morphological differences between *S*. *cavum* and *S*. *yuphae* ‘A’ are slight: number of columns of upper-eye facets in males and size of the tubercles on the pupal frons [18]. Either *S*. *cavum* is conspecific with *S*. *yuphae* ‘A’, in which case the morphological differences represent intraspecific variation and *cavum* becomes a synonym of *yuphae*, or *S*. *cavum* and *S*. *yuphae* are homosequential species [38–40]. *Simulium yuphae* ‘B’ was identified morphologically as a new species. Chromosomally, however, the only evidence for species status, separate from *bona fide S*. *yuphae* ‘A’, which was collected about 800 km to the south, was a potentially differentiated sex chromosome in the two male larvae in our sample. The hypothesis that IIIL-57 and IIIL-58 are sex (Y?) linked in ‘B’ requires testing.

Agreement among chromosomal, molecular, and morphological taxonomic divisions is encouraging, but the discrepancies argue for closer scrutiny and an integrated approach [41,42]. For instance, not all cytoforms in the *S*. *tani* complex in Thailand can be evaluated for species status based on chromosomal evidence alone [19]. Molecular evidence, however, suggests that Thai cytoforms ‘A’, ‘C’, and ‘G’ are merely cytotypes—polymorphic members of a single species in an early stage of differentiation [13]. Discrepancies between cytogenetic and molecular analyses have been found in other Oriental members of the *S*. *tuberosum* group. *Simulium weji*, for example, has low cytogenetic diversity, suggesting a single species, but high molecular genetic diversity that partitions into groups of possible cryptic species [43].

### Phylogenetic relationships

Phylogenies based on polytene chromosomes can provide excellent topological agreement with those based on nucleotide sequence data, and can even be richer in information [33,44]. One of the most strongly supported phylogenetic relationships is the split of the *S*. *tani* lineage from all other members of the Southeast Asian *S*. *tuberosum* group [33,34]. A molecularly inferred phylogeny of the Oriental *S*. *tani* complex indicates that taxa cluster according to geography; thus, members of the complex are arranged in four monophyletic groups corresponding to Malaysia, Taiwan, Thailand, and Vietnam [13]. The chromosomally inferred phylogeny for the *S*. *tani* complex, however, does not show country fidelity of clades.

As with the limited set of morphological characters available in the Southeast Asian *S*. *tuberosum* group [45], a dearth of shared chromosomal characters also limits the extent to which phylogenetic relationships can be inferred. Although certain chromosomal synapomorphies (e.g., *IIIL-11*, *IIIL-13*) provide a strong phylogenetic signal, the scarcity of shared rearrangements for taxa in Vietnam, coupled with the challenge of determining if they are derived (i.e., by comparison of the often-scrambled sequences against the sequences in outgroups), can limit their utility. An integrated approach that taps the potentially larger set of molecular characters [13] will be needed for a fully resolved phylogeny.

### Chromosomal and taxonomic biodiversity

The chromosomal rearrangements discovered in our Vietnamese samples bring to 180 the number now known for the Asian *S*. *tuberosum* group. These 180 rearrangements are distributed among 40 cytologically distinct taxa, 38% of which are known from Vietnam. Intra- and interspecific inversions are disproportionately concentrated (>50%) in the IIIL arm, not only for taxa in Vietnam, but also for the entire *S*. *tuberosum* group in the Palearctic and Oriental Regions [19,24,30,46]. The highly labile nature of IIIL suggests that the arm is given to increased fragility, that the retention rate of the breakage products (i.e., inversions) is higher, or both. In contrast, not a single rearrangement is known from the IIIS arm in the Oriental Region, other than the displaced nucleolar organizer, which is a synapomorphy for the entire *S*. *tuberosum* group [19]. The Nearctic members of the group express the majority of their interspecific chromosomal differences in IIS rather than IIIL, reflecting an independent evolutionary trajectory sometime after divergence from the ancestor of the *S*. *tuberosum* group [22,47].

The discovery of 88 different rearrangements, beyond the 25 characteristic of the basic sequences, among 272 Vietnamese larvae contrasts sharply with the 50 rearrangements found among 3347 Thai larvae [19]. The number of cytoforms discovered in Vietnam (15), however, is roughly the same as that known in Thailand (16). Chromosomal comparisons of the four nominal species shared between Vietnam and Thailand (*S*. *tani*, *S*. *doipuiense*, *S*. *rufibasis*, and *S*. *yuphae*) reveal the same trend—a greater number of different rearrangements for each of the four nominal taxa in Vietnam, when corrected for sample size, despite fewer to roughly the same number of sampling sites in about the same number of ecoregions (4 or 5) (*sensu* [48]). These rearrangements represent 2.0–2.5 times more cytoforms of each of these four taxa in Vietnam, except *S*. *tani*, which consists of 2.3 times more cytoforms in Thailand where the number of its analyzed larvae was 25 times greater than in Vietnam. The high chromosomal diversity in Vietnam, thus, is not an artifact of sampling, lending credence to the country’s status as a biodiversity hotspot.

The diversity of rearrangements and the taxa they represent in Vietnam overlaps minimally with that in Thailand. Only two cytoforms (*S*. *doipuiense* ‘A’ and *S*. *yuphae* ‘A’) and seven rearrangements (IL-2, IIIL-5, *IIIL-8*, *IIIL-11*, IIIL-12, *IIIL-13*, and *IIIL-34*), beyond the basic sequences, are shared between Thailand and Vietnam. The minimal congruence might reflect distance, local adaptation, and periods of population isolation resulting from glacial cycles. The ecological diversity among Oriental members of the *S*. *tuberosum* group has been suggested as an indication that ecological adaptation has played a role in driving evolution in the group [33,43,49]. The *S*. *tuberosum* group in Southeast Asia is found in the mountains, which has promoted population divergence in other black flies, with the intervening lowlands restricting gene flow [50]. The isolation of populations at higher elevations would have been particularly acute during glacial periods when tropical areas were drier and streams flowed only in high mountains [49].

More generally, the *S*. *tuberosum* group has 10 nominal species in the Nearctic Region, 23 in the Palearctic, and 32 in the Oriental, with 8 of these in Thailand, 10 in Malaysia, and 14 in Vietnam [21,51], plus 1 additional valid, but unnamed, species in Vietnam revealed in our study. Thailand is 1.5 times larger than Malaysia and Vietnam and has been intensively surveyed for simuliids since 1984 [52]. In contrast, focused simuliid exploration began in Vietnam only in 2014 and has been restricted to a limited portion of the country [15–18,51]. The only comparable analyses of chromosomal diversity in the group beyond the Oriental Region have been conducted in eastern Canada (Nearctic Region) and Hokkaido, Japan, plus two provinces in South Korea (Palearctic Region). Analysis of 1190 larvae (350 larvae for the IS arm) from eastern Canada revealed five cytoforms and 93 different rearrangements [22], whereas 118 larvae from Japan and Korea revealed three cytoforms and 28 different rearrangements [24]. Correcting for sample size, the number of different rearrangements per larva remains greater for Vietnam: 0.32 versus 0.24 for Japan plus Korea, 0.06 for eastern Canada, and 0.01 for Thailand.

Speciation in the Simuliidae has been associated with chromosomal phenomena, particularly coadaptation of sex chromosomes [37], cooption of individual rearrangements for different roles (e.g., fixation, X linkage) in different lineages, and more rarely, larger genomic restructuring events such as translocations [36]. Of the 15 Vietnamese cytoforms, four or fewer (*S*. *brevipar* ‘B’, *S*. *doipuiense* ‘E’, *S*. *rufibasis* ‘B’, and *S*. *yuphae* ‘B’) have differentiated sex chromosomes, compared with seven of 16 in Thailand [19]. At most, only three pairs of Vietnamese taxa provide examples of a differentially expressed rearrangement: (1) *Simulium tani* ‘B1’ and ‘B2’ are defined on the basis of IL-2, which is polymorphic or fixed in ‘B1’ and absent in ‘B2’, (2) the *S*. *yuphae* complex and *S*. *rufibasis* ‘B’ carry IIIL-12, which is fixed in the former and polymorphic in the latter, and (3) *S*. *doipuiense* ‘A’ and ‘E’ have heteroband 100hb1, the former as an autosomal polymorphism and the latter as an X-linked rearrangement. This situation contrasts with the pattern in the *S*. *tani* lineage in Thailand [19] and numerous other groups of simuliids [26,53] in which closely related cytoforms are defined in whole or in part by the same rearrangement operating in as many as five different roles. Thus, diversification of simuliids in Vietnam, although corresponding in part to general patterns of chromosomal restructuring associated with speciation, might include additional chromosomal phenomena or altogether different phenomena, such as those operating at the molecular level.

The discovery of hidden diversity in the *S*. *tuberosum* group in Vietnam follows an emerging pattern in the Simuliidae—cryptic diversity is not uniformly distributed across taxa. Rather, certain nominal (morphologically based) “species” in each zoogeographic region have a disproportionately greater degree of cryptic diversity. Diversity-rich taxa, formerly considered single species, include *Helodon onychodactylus* and *S*. *arcticum* in the Nearctic Region [54–56], *Simulium metallicum* in the Neotropical Region [57], *Simulium colombaschense* in the Palearctic Region [25], and the super-rich *Simulium damnosum*—the largest species complex of blood-feeding arthropods in the world—in the Afrotropical Region [58]. *Simulium tani* and *S*. *doipuiense* represent this pattern in the Oriental Region. As additional morphospecies are screened for cryptic biodiversity, attention to this trend should provide insights into the process(es) responsible for uneven cryptic diversification.

## Conclusions

Comparative analyses indicate that chromosomal and species diversity in the *S*. *tuberosum* group is greatest in the Oriental Region, particularly in Vietnam. Our samples of the 15 cytoforms in Vietnam show a typical right-skewed distribution of relative abundance, with one abundant cytoform (*S*. *doipuiense* ‘A’) and five others represented by one or two individuals. The implication of the high proportion (33%) of rare cytoforms is that increased sampling across space and time would reveal additional taxa. The urgency is increasing to discover the extent of biodiversity in Vietnam before it is too late. The montane forests that provide suitable habitat for the *S*. *tuberosum* group and other taxa in Vietnam are under threat from the pressures of a rapidly growing human population [4] that now approaches 100 million. Given the restricted geographical distributions suggested by our findings, some taxa, including those not yet discovered, could be imminently threatened.
